# Scaling up Family Medicine and Primary Health Care in Africa: Statement of the Primafamed network, Victoria Falls, Zimbabwe

**DOI:** 10.4102/phcfm.v5i1.507

**Published:** 2013-03-28

**Authors:** Jan De Maeseneer

**Affiliations:** 1 Department of Family Medicine and Primary Health Care, University of Ghent, Belgium

From 21 to 23 of November 2012, participants from 20 countries convened at the Fifth Annual Primafamed Conference (www.primafamed.ugent.be) at Victoria Falls, Zimbabwe. The participants want to support fully the realisation of the World Health Assembly (WHA) resolution 62.12^1^, by contributing:

… to train and retain adequate numbers of health workers, with appropriate skill-mix, including primary health care nurses, midwives, allied health professionals and family physicians, able to work in a multidisciplinary context, in cooperation with non-professional community health workers in order to respond effectively to people’s health needs. 

The participants recognise the importance of the worldwide demographic and epidemiological transitions and the impact of the global economic crisis on health and that these phenomena give rise to new challenges for healthcare providers in Africa. Moreover, the participants stress the need for an integrated approach to comprehensive PHC in order to address the fragmentation of care and health systems as a consequence of vertical disease-oriented programmes (HIV, malaria, COPD, diabetes, etc.). They confirm their commitment to the realisation of the WHA resolution 62.12:^1^

… to encourage that vertical programmes, including disease-specific programmes, are developed, integrated and implemented in the context of integrated primary health care,

the WHO Global Health Workforce Strategy^2^ and the WHA resolution 59.23: ‘Rapid Scaling Up of Health Workforce.’^3^ 

Family physicians fully support African governments’ implementation of universal health coverage that is oriented towards guaranteeing the right to health for all. This includes implementing the Abuja Declaration of allocating 15% of budget to health; developing national socially oriented health insurance systems to provide universal access; single risk pools and adjusted capitation systems to ensure that resources go to those who need it most; strong decentralised district health systems responsive to local communities; the inclusion and regulation of the private for-profit and not-for-profit sectors as contracted providers; and innovative payment systems to drive quality improvement in integrated primary healthcare teamwork to achieve ‘Health for All’.

The participants define the future of family medicine (FM) in the framework of the PHC system: a community-based team approach that includes nurses, family physicians, mid-level care workers (associate clinicians), health promoters and community health workers; an approach that focuses on accessibility, connectedness, health promotion and disease prevention, comprehensiveness, continuity, and coordination, in the context of families and communities (as described in the Consensus Statement, Rustenburg 2009).^4^

Family physicians in Africa take responsibility for specific tasks in the district and in district or community hospitals, and need to be trained accordingly. The training of family physicians has to take place mainly in an inter-professional PHC team context in the district health system. Family physicians share responsibility for training other health care workers in PHC.

Increasingly, PHC will have to address the problems arising from the context of multi-morbidity, and provide appropriate person- and people-centred care. Community oriented primary care (COPC) is an appropriate strategy embraced by African PHC teams to address upstream causes of ill-health, including behavioural, social and environmental determinants.

The departments and training institutions for FM commit to a socially accountable approach in order to respond to the workforce needs and the requirements of the health care system. Training in family medicine is based on the acquisition of appropriate knowledge, skills and attitudes in the context of the community, with an emphasis on community-based training in the programmes.

In terms of the scaling up of FM, a reflection is ongoing on the optimal duration of postgraduate training, which will vary in relation to the relevance of the undergraduate training and contextual factors such as the need to work in the district or community hospital. In a study on the principles of FM in Africa the need to perform common clinical procedures and operations appropriate to the district health system was a reality in 70% of the settings.^5^ One example that inspired the participants was the report of a two-year training programme in the community that was implemented by the Faculty of Medicine of Gezira University and the Ministry of Health in Sudan. It addressed the needs of the local population, was supported by e-learning, and involved 200 candidates. This is an example on which we need to reflect in order to develop optimal training programmes in each country. The reflection will include a determination of the need to learn procedural skills (surgical, anaesthetic, etc.) in each country. These and other topics will be addressed in the process of mutual learning and exchange in the Primafamed network.

In order to scale up FM, concrete strategic actions should be developed, including the following:

- Convince Ministers of Health and Education as well as the leadership at medical schools that a significant proportion of the graduates (between 40% and 60%) should be trained in FM and PHC.
- Integrate the existing community service period into the FM training programme, in order to fast-track the scaling up at a lower cost.
- Define the appropriate content and duration of the training programme in each country.
- Prepare for lifelong learning and develop appropriate Continuous Professional Development.

The following are essential conditions to make this happen:

- Ensure that all countries have training in family medicine.
- Establish networks, synergies and collaborations to support African standards. 
- Integrate exposure to PHC and FM in the undergraduate curriculum and provide role models for FM. 
- Establish well-equipped training complexes for PHC teams (PHC centres and related clinics, with the district hospital for referrals), creating an environment for transformative learning. 
- Offer sufficient funded posts for residents or registrars. 
- Provide appropriate remuneration for family physicians and PHC-teams and attractive career paths.
- Develop train-the-trainer programmes, taking advantage of South-South cooperation. 
- Increase the budget for PHC.
- Encourage NGOs and donors to invest in strengthening local PHC-systems (www.15by2015.org). 
- Implement population-oriented campaigns to promote FM and PHC and stimulate cost-effective use of health care services by the population.

If these conditions were fulfilled from today onwards, it will be possible to train 30 000 new family physicians and tens of thousands of PHC professionals for PHC teams in sub-Saharan Africa in the next ten years. In the sub-Saharan African Medical School study the authors calculated that the data suggest an estimated 10 000–11 000 graduates per year from medical schools in sub-Saharan Africa.^6^ If the hypothesis that 50% of the graduates would be trained in Family Medicine from 2013 onwards becomes a reality, this would lead to 30 000 new Family Physicians in sub-Saharan Africa by 2020 in two-year programme and by 2022 in a four-year programme (at higher cost). In Europe, the required post-graduate training for family medicine is three years.^7^

The participants stress that appropriate research in FM and PHC in Africa is essential, in order to substantially enlarge the evidence base for the issues highlighted in this document. This should be facilitated by the provision of specific funding by governments and Non Governmental Organisation (NGO), by building the research capacity in academic departments of FM and PHC, and by developing an African FM and PHC research network to support researchers and promote cross-country collaboration.

The delegates from the Primafamed-Network Conference want to engage in a regional strategy through dialogue at different levels: local level, the community, stakeholders, provinces, and national government (Ministers of Health). Moreover, at the international level they call upon the World Health Organisation-Africa region, African Union, Wonca, Medical East Partnership Initiative and other organisations to strengthen their commitment to the development of PHC and FM. 

If this takes place, the delegates are convinced that they can make a difference where it really matters, to contribute to a healthier future for Africa.

Participants at the Primafamed Network-conference 2012 came from the following African countries: Botswana, Ethiopia, Ghana, Kenya, Lesotho, Malawi, Mozambique, Namibia, Nigeria Rwanda, South Africa, Sudan, Swaziland, Uganda, Zambia, Zimbabwe. There were also participants from Belgium, Canada, Denmark, England, Ireland, Norway, The Netherlands, Scotland, and the USA.

## References

[1] Sixty-second World Health Assembly. Primary health care, including health system strengthening [homepage on the internet]. 2009 [Cited 2012 Dec 15]. Available from http://www.who.int/hrh/resources/A62_12_EN.pdf

[2] World Health Organization. Workshop on global health workforce strategy [homepage on the internet]. 2000 [Cited 2012 Dec 15]. Available from http://www.who.int/hrh/documents/en/workforce_strategy.pdf

[3] World Health Organization. WHA 59.12 Rapid scaling up of health workforce production [homepage on the internet]. 2006 [Cited 2012 Dec 15]. http://www.who.int/workforcealliance/knowledge/resources/wha_scalingup/en/index.html

[4] Mash B, Reid S Statement of consensus on Family Medicine in Africa. Afr J Prm Health Care Fam Med 2010;2 (1), Art. #151, 4 pages. doi: 10.4102/phcfm.v2i1.151

[5] Mash R, Downing R, Moosa S, De Maeseneer J Exploring the key principles of Family Medicine in sub-Saharan Africa: International Delphi consensus process. SA Fam Pract 2008;50 (3):60-65. www.safpj.co.za

[6] Mullan F, Frehywot S, Omaswa F, Medical schools in sub-Saharan Africa. Lancet 2011;377 (9771):1113-1121. doi: 10.1016/S0140-6736(10)61961-7 21074256

[7] European Parliament and the Council of the European Union. Directive 2005/36/EC [homepage on the internet]. 2005 [Cited 2012 Dec 15]. Available from http://eur-lex.europa.eu/LexUriServ/LexUriServ.do?uri=OJ:L:2005:255:0022:0142:en:PDF

